# Evaluation and comparison of unsupervised methods for the extraction of spatial patterns from mass spectrometry imaging data (MSI)

**DOI:** 10.1038/s41598-022-19365-4

**Published:** 2022-09-20

**Authors:** Mridula Prasad, Geert Postma, Pietro Franceschi, Lutgarde M. C. Buydens, Jeroen J. Jansen

**Affiliations:** 1 IMM/Analytical Chemistry, Radboud University, Heyendaalseweg, 6525 AJ Nijmegen, The Netherlands; 2 Unit of Computational Biology, Research and Innovation Center, Fondazione Edmund Mach, 38010 San Michele all’ Adige, Italy

**Keywords:** Functional clustering, Statistical methods

## Abstract

For the extraction of spatially important regions from mass spectrometry imaging (MSI) data, different clustering methods have been proposed. These clustering methods are based on certain assumptions and use different criteria to assign pixels into different classes. For high-dimensional MSI data, the curse of dimensionality also limits the performance of clustering methods which are usually overcome by pre-processing the data using dimension reduction techniques. In summary, the extraction of spatial patterns from MSI data can be done using different unsupervised methods, but the robust evaluation of clustering results is what is still missing. In this study, we have performed multiple simulations on synthetic and real MSI data to validate the performance of unsupervised methods. The synthetic data were simulated mimicking important spatial and statistical properties of real MSI data. Our simulation results confirmed that K-means clustering with correlation distance and Gaussian Mixture Modeling clustering methods give optimal performance in most of the scenarios. The clustering methods give efficient results together with dimension reduction techniques. From all the dimension techniques considered here, the best results were obtained with the minimum noise fraction (MNF) transform. The results were confirmed on both synthetic and real MSI data. However, for successful implementation of MNF transform the MSI data requires to be of limited dimensions.

## Introduction

Mass spectrometry imaging (MSI) is a valuable molecular imaging technique that provides a spatial distribution of several molecular ions present in a biological sample^1^. High-dimension MSI data provides an unprecedented opportunity to understand the molecular changes in the biological system in association with their spatial locations^2–4^. Depending upon the ionization mode and mass spectrometer used, MSI can analyze small to very large molecules which makes it a desirable technique in biology^5–9^ and medicine^10–13^.

MSI data are often organized in a three-dimensional cube where the spatial (x, y) dimensions correspond to the sample dimensions, while a spectral (z) dimension corresponds to the m/z (mass-to-charge ratio) values measured by the mass spectrometer. Most commonly, MSI data is produced or analyzed either to get knowledge about spatial localization of important and known molecular ions^5,7–9,14^, in a so-called “targeted” approach or to identify spatially relevant regions^15–21^. For the identification of spatially relevant regions, the complete MSI data need to be analyzed simultaneously with little or no prior information about a biological sample. Therefore, unsupervised data analysis methods, such as clustering, provide a good solution to extract hidden patterns in the data. A variety of clustering methods has already been implemented on MSI data and has been proven to efficiently extract relevant clusters equivalent to biological structures^17–19,22–25^. All these clustering methods are based on certain assumptions regarding data structure and use different criteria to group the mass spectra into different classes.

In the last few years, MSI technology has gone through constant development in terms of spatial resolution^26–28^, mass accuracy^26,29,30^, sample preparation^31,32^, etc. to produce high-quality and reproducible data^33–35^. The performance of clustering methods decreases as data size increases in spectral dimension due to curse-of-dimensionality. To overcome this problem, clustering methods are usually combined with dimension reduction techniques^23^. Dimension reduction is important in cluster analysis because it not only makes the high dimensional data addressable but can also provide users with a clearer picture and visual examination of the data of interest. Mc Combie et al.^16^ were efficiently able to cluster the MSI data using hierarchical clustering on the reduced space obtained after principal component analysis (PCA). t-SNE is another popular multidimensional data visualization technique^36,37^ that is frequently combined with clustering methods on MSI data, especially in cancer research to find intra-tumor heterogeneous subpopulations^18,23^. In addition to this, several other dimension reduction techniques were proposed for MSI data^38^. In summary, there are several unsupervised methods suggested to extract spatially important regions from MSI but it is still unclear which method gives the optimal performance. Only one single study tries to validate the performance of k-means clustering with different similarity/dissimilarity measures^39^ on MSI data. Unfortunately, this study focuses on one type of clustering method and the synthetic data used only reflect a difference in metabolic profile between the different clusters. This simplified structure is not able to capture the characteristics of “real” MSI data which show correlation both in the spectral and spatial domain, Therefore, in this paper we compare the state-of-art clustering methods in combination with dimension reduction techniques taking into account possible spatial characteristics, to find the most appropriate method for clustering MSI data and hence for the identification of spatial patterns. Multiple simulations were performed on synthetic and real MSI data to validate the performance of the various clustering methodologies.

## Material and methods

MSI datasets were used for the evaluation of clustering methods obtained from online published research.

### Mass spectrometry imaging data1

The MSI data were obtained from a tissue section of a mouse urinary bladder^29^ and are publicly available from the PRIDE^40^ repository (PXD001283) managed by the European Bioinformatics Institute (EBI). The mouse urinary bladder was sectioned in 20 µm thickness slices with a cryotome (HM500, Microm, Walldorf, Germany) and transferred to a conductive ITO-coated glass slide. DHB (2, 5-dihydroxybenzoic acid) matrix was applied using a pneumatic sprayer and an AP-SMALDI imaging source was used. The imaging source was attached to a linear ion trap/Fourier transform orbital trapping MS (LTQ Orbitrap Discovery, Thermo Scientific GmbH, Bremen, Germany). A UV laser with a repetition rate of 60 Hz (LTB MNL-106, LTB, Berlin, Germany) was used for desorption/ionization. The mass resolving power was 30 000 at m/z 400 in positive-ion mode. Matrix-assisted laser desorption/ionization (MALDI) images were acquired using a pixel size of 10 µm in both x and y directions and an m/z range of 400–1000 Da. The dataset comprises 34,840 spectra acquired within the slice area (260 × 134 pixels). The experimental details regarding sample preparation and data acquisition for particular MSI data are given in^29^.

### Mass spectrometry imaging data2

The MSI data is publicly available in the GigaScience repository, GigaDB^41^. The MSI data were derived from tumor-bearing mice treated with paclitaxel drug. A matrix-assisted laser desorption/ionization (MALDI) 4800 TOF-TOF (AB SCIEX, Framingham, MA) was used. The mass spectra were recorded over a limited mass range (m/z 199–500). The mass spectra were collected from a glass slide of dimension 106 × 85. More details regarding sample preparation and MSI data acquisition are given in^41,42^.

### MSI data preprocessing

All data preprocessing steps were performed in R^43^ free software version 3.1. The original MSI data file was read in R using the MALDIquant^44^ package and organized in a two-dimension matrix where the row represents the complete mass spectrum collected from individual spatial locations. The preprocessing of MSI data was done following the steps mentioned in article^41^. Briefly, the binning of the m/z dimension (bin of size 0.1 Da) was performed to compensate for misalignment on the m/z scale. The peak detection was performed inside each bin per mass spectrum. In the case of multiple peaks detection inside the bin, the peak with maximum intensity is stored. The peak detection was performed using a local maxima search. The identification of tissue over the glass slide was performed by constructing a mask of the ion signal selected after visual inspection The matrix-associated peaks were identified from the spectra collected outside the tissue and removed before further analysis. Total ion current normalization was performed to compensate for analytical pixel-to-pixel variability and make mass spectra comparable with each other. In addition, median filtering per mass ion image was performed to reduce pixel-to-pixel variability.

In the Mouse Urinary Bladder MSI data, the final data dimension achieved after all preprocessing steps was 260 × 134 × 169. And, for the tumor tissue was 106 × 85 × 173.

### Synthetic spatially auto-correlated data

The spatial data were simulated to evaluate the performance of clustering methods. In our simulated data, the spatial autocorrelation was induced using a variogram or spatial covariance function^45^.

### Theory

*Variogram* A variogram is a plot of semi-variance versus spatial lag distance that describes the degree of spatial dependence between measurements at sample locations. An experimental variogram is calculated based on the sample data as:

$$r\left( h \right) = \frac{1}{2N\left( h \right)}\mathop \sum \limits_{i = 1}^{N\left( h \right)} \left[ {Z\left( {x_{i} } \right) - Z\left( {x_{i} + h} \right)} \right]^{2}$$

where $r\left( h \right)$ = the variogram for a lag distance $h$ between observations $Z\left( {x_{i} } \right)$ and $Z\left( {x_{i} + h} \right)$, $h$ = the distance between sample intervals, $N\left( h \right)$ = the number of data pairs separated by lag distance $h$, $Z\left( {x_{i} } \right)$ = the value of variable $Z$ at the location of $x_{i}$, $Z\left( {x_{i} + h} \right)$ = the value of $Z$ located at a lag distance of $h$ from $x_{i}$.

**Figure 1:**
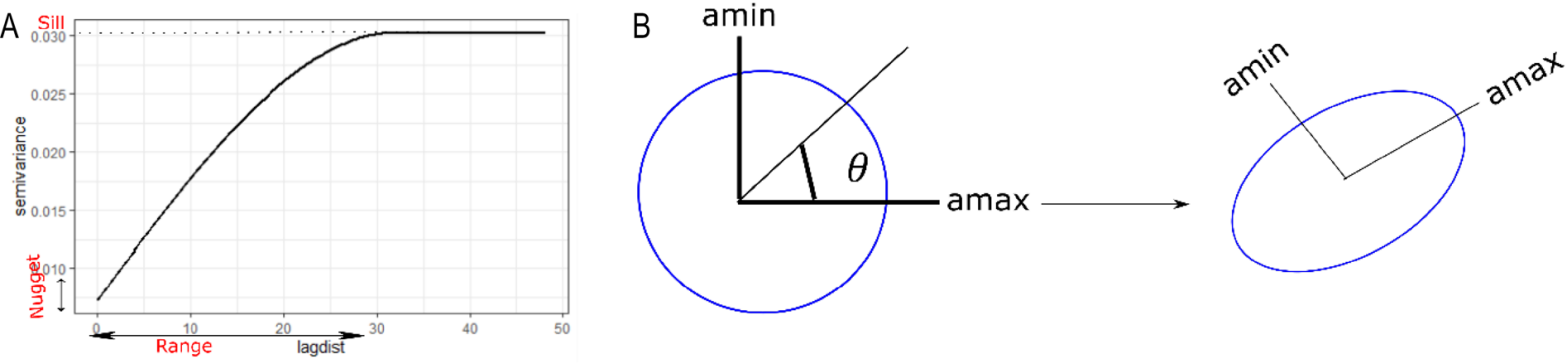
(**A**) Variogram plot derived from spatial data. A variogram shows the amount of spatial autocorrelation in the data where the parameter ‘Range’ indicates the distance between two observations beyond which observations appear independent. The ‘sill’ is the point at which semi -variance reaches an asymptote. The nugget is the spatial variability at the origin. The variogram plot is created using R-package gstat^62^. (**B**) The conversion of isotropic spatial coordinates to anisotropic spatial coordinates. The isotropic spatial data has the same amount of spatial autocorrelation in all directions while in anisotropic data particular direction has stronger autocorrelation than other directions. Isotropic data can be converted into anisotropic data using rotation and scaling matrix (see “Material and methods”).

A standard variogram is shown in Fig. 1A. Three key parameters are estimated from an experimental variogram to fit a theoretical variogram: nugget, sill, and range. The nugget is the spatial variability at the origin or zero sampling interval. The range indicates the maximal distance at which the variable is spatially autocorrelated. As the distance lags increase, the semi-variance values rise continuously until it reaches a certain value called the sill.

*The covariance function* is another statistical measure to show spatial autocorrelation. In our paper we used the term variogram and covariance function interchangeably due to the simple relationship between them^45^:

$$r\left( h \right) = C\left( 0 \right) - C\left( h \right)$$

$C\left( 0 \right)$ is the value at the origin of the covariance function. $C\left( h \right)$ is the value at distance $h$ of the covariance function.

The commonly used variogram or covariance functions for modeling are spherical, gaussian, and exponential. In our study, we used the spherical covariance function:

$$C_{sph} \left( h \right) = \left\{ {\begin{array}{*{20}l} {b\left( {1 - \frac{3}{2} \frac{\left| h \right|}{{a }} + \frac{1}{2}\frac{{\left| h \right|^{3} }}{{a^{3} }}} \right)} \hfill & {for\; 0 \le \left| h \right| \le a} \hfill \\ 0 \hfill & {for\; \left| h \right| > a} \hfill \\ \end{array} } \right.$$

In the above formula, parameter $a$ is the range, $b$ is the sill, and $h$ is the lag distance. In our simulations described below, parameters $a$ and $b$ are modified to generate spatial data with different structures. $h$ is calculated from the Euclidean distance matrix of observations spatial coordinates.

*Anisotropy* The spherical covariance function mentioned above will generate the isotropic spatial data, i.e. the covariance between variables at two locations depends only on the distance between them and not on the direction. The geometric anisotropy^46^ can be introduced by rotating and rescaling the spatial coordinates (Fig. 1B).

$$c^{*} = c\left( {RT} \right)^{ - 1}$$

$c$: input matrix of coordinates in the isotropic space. $c^{*}$: output matrix of coordinates in the anisotropic space. $R$: Rotation matrix.

$$R = \left[ {\begin{array}{*{20}c} {{\text{cos}}\left( \theta \right)} & {{\text{sin}}\left( \theta \right)} \\ { - {\text{sin}}\left( \theta \right)} & {{\text{cos}}\left( \theta \right)} \\ \end{array} } \right]$$

$\theta$ is the angle of rotation. $T$: Scaling matrix.

$$T = \left[ {\begin{array}{*{20}c} {a_{max} } & 0 \\ 0 & {a_{min} } \\ \end{array} } \right]$$

$a_{max}$, $a_{min}$ are a major and minor range of anisotropy ellipse.

### Synthetic spatial data generation

For the evaluation of clustering methods, two types of spatial data were generated, i.e., with two and four clusters. The spatial data of dimension 80 × 80 × 100 was simulated where 80 pixels in both x and y directions, and 100 variables. The dimensions of the individual cluster in the synthetic data with two clusters were 80 × 40 × 100 and in four clusters data were 80 × 20 × 100 .

The initial 100 variables with a mean of zero and standard deviation of one were derived from different distribution types: normal, non-normal, and bimodal. To add correlation among those independent variables, a cluster-wise correlation matrix was generated using the MixSim^47^ R package. In our correlation matrix, the range of correlation varied between 0.4 and 0.7. The correlation matrix was decomposed into a triangular matrix using the Cholesky method and the upper triangular matrix was then used to add the correlation among independent variables.

A spatial covariance matrix was designed and used to convert multivariate correlated data into spatially autocorrelated data. A single large spatial covariance matrix is made up of multiple spatial covariance matrices equal to the number of clusters in spatial data. Inside the spatial covariance matrix, the parameters for the spherical covariance function were modified to get unique clusters. Before, creating a spatial covariance matrix an anisotropic effect was added using the above-mentioned formula to avoid randomness in the data. For the anisotropic effect, the parameters used were the rotation angle of ${45}^{o}$ and, the major and minor range of one and 0.5 in the scaling matrix.

A single complete spatial covariance matrix was generated by arranging the spatial covariance matrices from the individual clusters using the process described here^48^. Similar to the metabolic correlation, for the spatial correlation effect the spatial covariance matrix was decomposed using the Cholesky method, and the upper triangular matrix is multiplied with multivariate correlated data. The schematic workflow of the data generation steps is summarized in Supplementary Fig. 1.

### Unsupervised methods

Four clustering and dimension reduction techniques were selected in our study to test on synthetic and real MSI data. The clustering methods selected were K-means with Euclidean distance (k-means (E)), K-means with correlation distance (k-means(C)), Spatially aware structurally adapted (SASA)^25^, and Gaussian mixture modeling^49^ (GMM).

All clustering methods were selected based on their frequency of usage on MSI data and/or ability to identify clusters of different shapes. The performance of clustering methods was validated using the adjusted rand index^50^ (ARI). An ARI evaluates the association between actual cluster class labels and the one obtained from the clustering methods. Clustering methods are implemented using different R libraries: stats^43^, amap^51^, mclust^50^, and cardinal^52^.

The dimension reduction techniques tested in our study were: Principal component analysis^38^ (PCA), Spatial principal component analysis^53^ (sPCA), Minimum noise fraction^54^ (MNF) transform, and t-Distributed Stochastic Neighbor Embedding^55^ (t-SNE). t-SNE is a non-linear multidimensional data visualization technique but in our paper, we referred to it under the dimension reduction technique since we used few dimensions for clustering. The dimension reduction techniques were performed using the following R packages: stats^43^, adegnet^56^, mzImage^54^, and Rtsne^57^. A brief description of clustering and dimension reduction techniques is given in Supplementary Text S1.

### Simulation study

**Figure 2:**
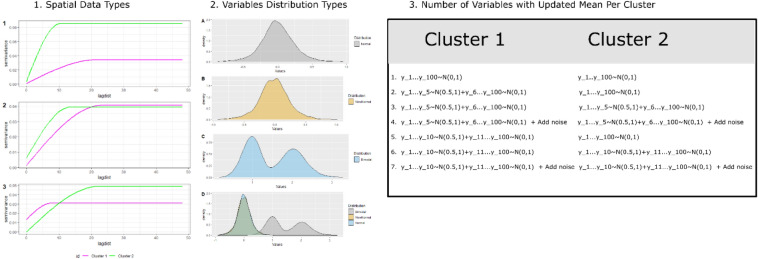
The three main categories for synthetic data generation in a simulation study. (**1**) The synthetic data with different spatial structures were generated using different input in variogram function (spatial data1: range1 = 20, range2 = 20, sill1 = 0.1, sill2 = 0.3; spatial data2: range1 = 20, range2 = 10, sill1 = 0.1, sill2 = 0.1; spatial data3: range1 = 20, range2 = 10, sill1 = 0.1, sill2 = 0.3). (**2**) The variables in synthetic data are derived from different statistical distributions: normal, non-normal, bimodal, and all combined. (**3**) The synthetic data was modified by increasing the mean value for a certain number of variables and adding the noise.

The performance of the clustering methods and dimension reduction techniques was investigated using a simulation study. In synthetic data, both spectral and spatial properties of variables were modified for the evaluation of clustering methods. In total 100 different simulations are performed in all possible combinations under the following three main categories (Fig. 2):

1. The type of spatial structure.

Three different types of spatial data were simulated by changing the value of range and sill parameters in the spatial covariance function. In spatial data type 1, both clusters are with same range (= 20) but with different sill value (sill1 = 0.1, sill2 = 0.3). In spatial data type 2, range in cluster is different (range 1 = 20, range2 = 10) but the sill is constant (sill = 0.1). And, in spatial data type3, both clusters are with different range (range1 = 20, range1 = 10) and sill (sill1 = 0.1, sill2 = 0.3) values. The range and sill for synthetic data with four clusters are mentioned in Supplementary Table S1.

2. The type of statistical distribution.

Four different scenarios are tested where variables in synthetic data are derived from different distribution types:

- scenario1 (normal distribution): all the variables follow a standard normal distribution with a mean of zero and a standard deviation of one.
- scenario2 (non-normal): all variables follow non-normal distribution simulated following Fleishman’s power method using SimMultiCorrData^58^ R package.
- scenario3 (bimodal): all variables follow bimodal distribution, i.e. approximately 20% of observations are from normal distribution1(N(0,1)) and remaining derived from normal distribution2 (N(1,0.2)).
- scenario 4 (all combined): out of 100, 60 variables follow a standard normal distribution, 20 variables follow the bimodal distribution, and the remaining 20 variables follow the non-normal distribution.

3. The variation in the mean value of variables.

There is a total of seven scenarios discussed under this category. The original variables in spatial data are derived from certain distributions with a mean of zero and a standard deviation of one. In the first scenario, there is no change in the variable mean. Afterward, the mean of a certain number of variables (5,10) increased by 0.5 first only in cluster 1 and then in both clusters. In addition, two more scenarios were tested with noise variables (5), i.e., variables without any spatial structure.

## Results

### Clustering results from the simulation study on synthetic data

**Figure 3:**
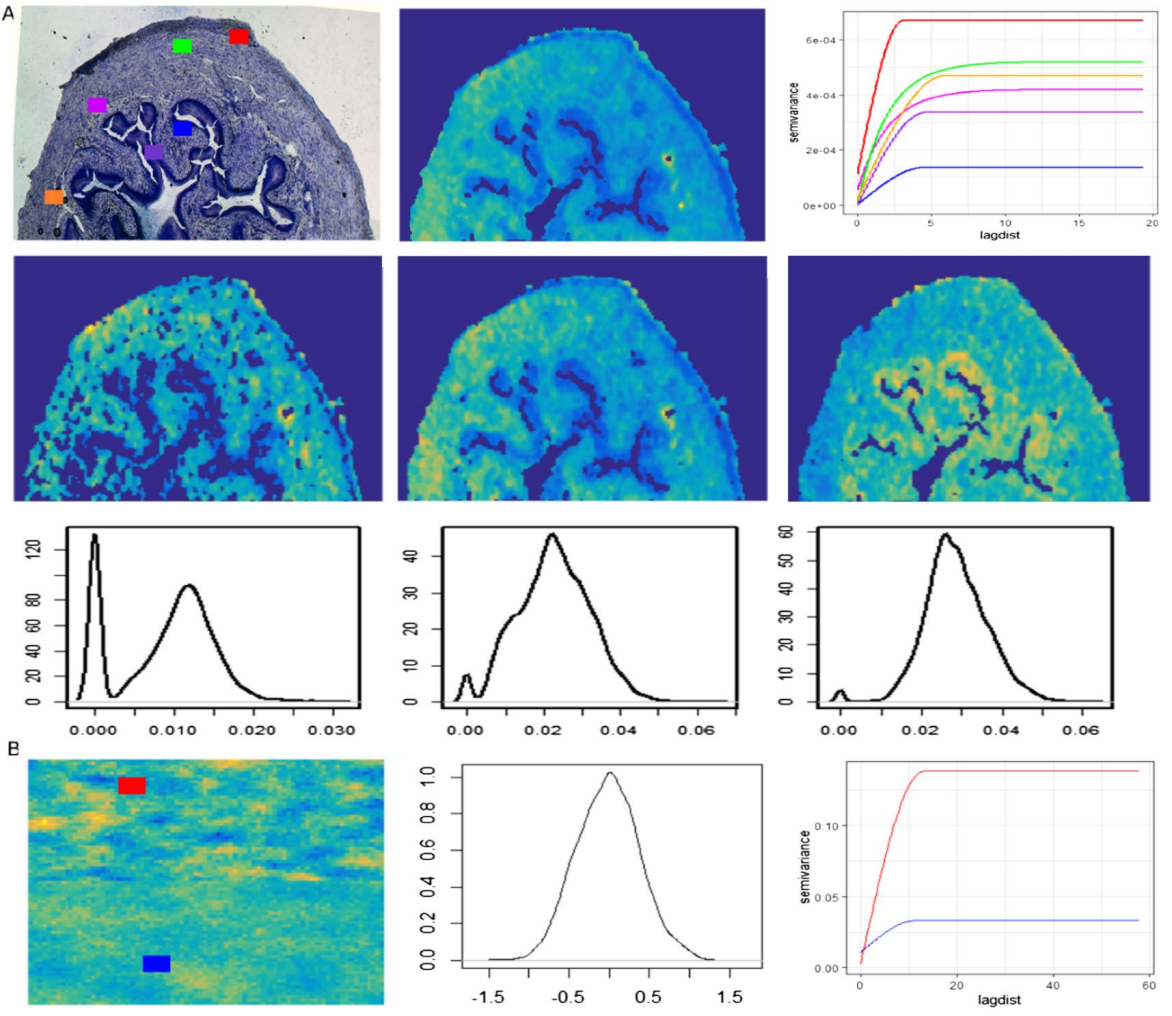
Comparison of statistical (distribution) and spatial (variograms) properties of variables from real MSI (a) and synthetic spatial data (b). (**A**) Top row: The variograms (right) of an example single ion (middle) from different regions are shown in its histology image (left). Middle row: the 2D image of a few molecular ions from real MSI data and their density plot in the bottom row. (**B**) The synthetic spatial data image (left), density plot (middle), and the variograms (right) from synthetic data. The variograms (left column) and density plots (middle column) from synthetic data.

Several simulations were performed to evaluate the performance of different clustering methods with and without data preprocessing methods. For simulation purposes, the synthetic spatial data was simulated after visualizing the spatial and statistical properties of different variables from the real MSI data (Fig. 3).

**Figure 4:**
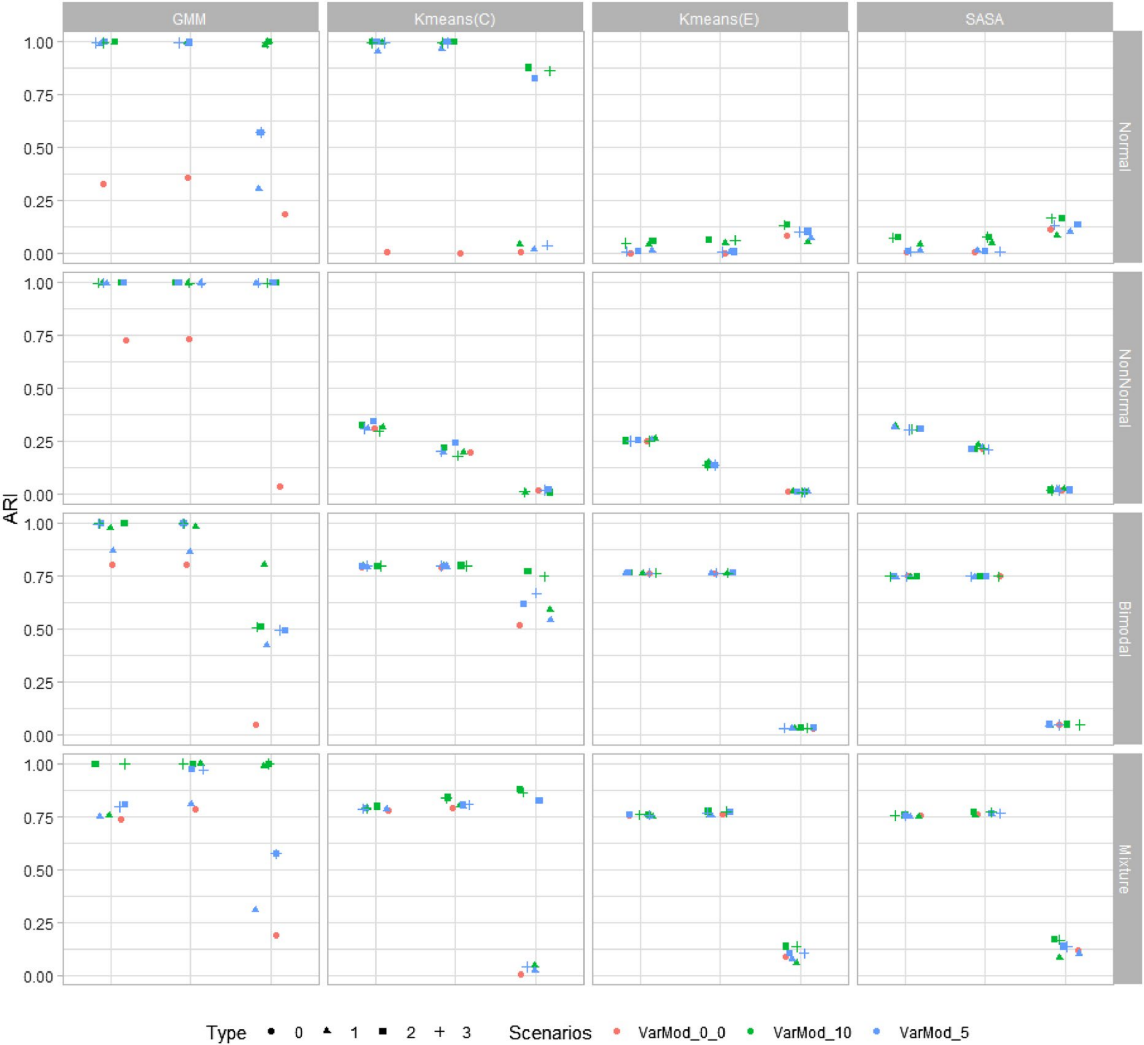
A plot of simulation results for the two clusters problem. All four clustering methods (GMM, K-means(C), K-means(E), and SASA) were tested on synthetic data with two clusters. The performance of clustering methods is monitored based on their adjusted rand index (ARI) value. In the above figure, the ‘Scenarios’ represent the number of variables with updated mean values in synthetic data, such as VarMod_0, all variables in synthetic data with zero mean; VarMod_10, 10 variables in synthetic data with mean value 0.5; VarMod_5, 5 variables in synthetic data with mean value 0.5. And the ‘Type’ represents the nested conditions with scenarios (0, all variables with mean 0; 1, variables with updated means in single cluster (VarMod_0_5, VarMod_0_10); 2, variables with updated means in both clusters (VarMod_5_5, VarMod_10_10); 3, simulation with added noise (VarMod_5_5_addnoise, VarMod_10_10_addnoise). The details of the statistical and spatial parameters modified are given in Fig. 2.

**Figure 5:**
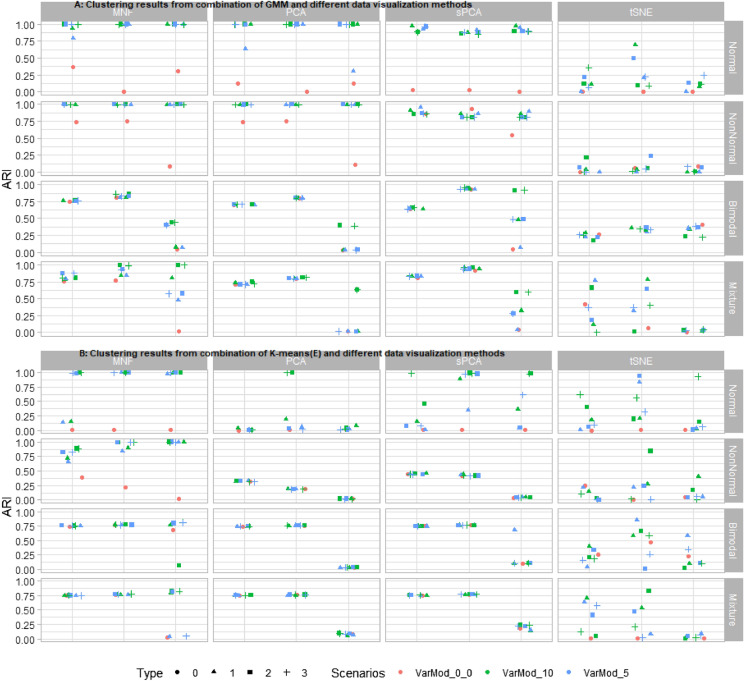
A plot of simulation results from the combination of dimension reduction techniques and clustering methods. Four different dimension reduction techniques (MNF, PCA, sPCA, and t-SNE) were implemented on synthetic data before cluster analysis. Cluster analysis was performed with (**A**) GMM and, (**B**) Kmeans(E) methods. The performance of clustering methods is monitored based on their adjusted rand index (ARI) value. In the above figure, the ‘Scenarios’ represent the number of variables with updated mean values in synthetic data, such as VarMod_0, all variables in synthetic data with zero mean; VarMod_10, 10 variables in synthetic data with mean value 0.5; VarMod_5, 5 variables in synthetic data with mean value 0.5. And the ‘Type’ represents the nested conditions with scenarios (0, all variables with mean 0; 1, variables with updated means in single cluster (VarMod_0_5, VarMod_0_10); 2, variables with updated means in both clusters (VarMod_5_5, VarMod_10_10); 3, simulation with added noise (VarMod_5_5_addnoise, VarMod_10_10_addnoise). The details of the statistical and spatial parameters modified are given in Fig. 2.

The variograms for a single molecular ion from different spatial regions show a different range of sill and range parameters (Fig. 3A top right). Similar patterns were observed for other ions. The conventional statistical properties of the data were explored via density plots for certain molecular ions. They show skewed, bimodal, non-normal, etc. types of distribution (Fig. 3A third-row). Finally, the standard benchmark synthetic data were simulated based on these observations. For example, the applied range and sill values are similar to those observed in real MSI data variograms (Fig. 3A). The conversion of non-spatial data into spatial data slightly modifies the original population density structure (Supplementary Fig. S2), but the density plots and the variograms for example variables show overall statistical properties (distribution type, spatial structure) are preserved (Fig. 3B). Below the simulations under different scenarios are discussed. All these simulations were performed to investigate the limitations of the different clustering and dimension reduction methods. The performance of the clustering methods was measured with the help of the adjusted rand index (ARI). A high ARI value for a clustering method implies that the method can recover the true underlying cluster structure. The median ARI value from different clustering methods over 100 simulations is shown in Figs. 4 and 5. The input parameters used with different dimension reduction techniques are mentioned in Table 1.

**Table 1:** Input parameter for different statistical methods.

Statistical methods	Type of input parameter	Input parameter value
PCA	Number of dimensions	4
sPCA	Number of dimensions	4
	Lag distance for spatial weight matrix	5
MNF	Number of dimensions	6
t-SNE	Number of dimensions	2
	Perplexity parameter	10
	Number of PCA dimensions	10

First, the synthetic data with two cluster classes were investigated (Fig. 4). In scenario one (first row in Fig. 4) with normally distributed variables, GMM and k-means(C) give the optimal performance for spatial data one and two and selectively for spatial data type three, i.e. for Var_Mod_10. Overall poor performance of k-means(E) and SASA was observed. In scenario two, where all variables follow a non-normal distribution, only GMM is efficiently able to identify the clusters. For spatial data with all variables following bimodal distribution, k-means(C) gives the most consistent and high performance on all spatial data types. The performance of k-means(E) and SASA were improved for spatial data types- one and two, because of the large difference in cluster means (Supplementary Fig. S1). Finally, in the last scenario, in which the synthetic data contain variables of all three types of distributions, all clustering methods give acceptable performance for spatial data types one and two. For spatial data type three, GMM and k-means(C) give optimal performance when data contains at least 10 variables with a mean larger than zero.

The effects of the various dimension reduction techniques were investigated for two clustering methods (GMM and k-means(E)). k-means(C) has not been investigated since in the reduced dimension space original variables are represented by orthogonal bases, therefore a proper correlation matrix can not be derived. The SASA clustering method is similar to k-means(E), except for the spatial part, and will give approximately similar results (this has been tested; results not provided). Hence, it is not included. In the majority of scenarios, the ARI values obtained from the clustering methods applied to the reduced data are higher than the ones obtained from the raw data (Fig. 5). The highest ARI values are observed when clustering methods are applied after the MNF transform. MNF transform improved the identification of the cluster even for complex data types, such as spatial data type three and scenario 2, in which the variables follow a bimodal distribution (for GMM). PCA and its spatial version improved the identification of the cluster, but not in all possible scenarios. The poor performance of clustering methods was observed after dimension reduction by t-SNE.

A similar analysis was repeated for the synthetic data with four clusters (Supplementary Fig. S3 and S4). The clustering results obtained from original and reduced data are quite similar to the ones obtained with two cluster problems. GMM gives optimal performance in most situations, followed by k-means(C). The k-means(E) and SASA give optimal results for very few scenarios. Finally, the GMM and k-means(E) clustering methods are applied to the reduced data obtained from different dimension reduction techniques. Similar to the two clusters problem, the highest ARI values are observed when clustering methods are applied to the MNF reduced space.

### Clustering results from the real MSI data

**Figure 6:**
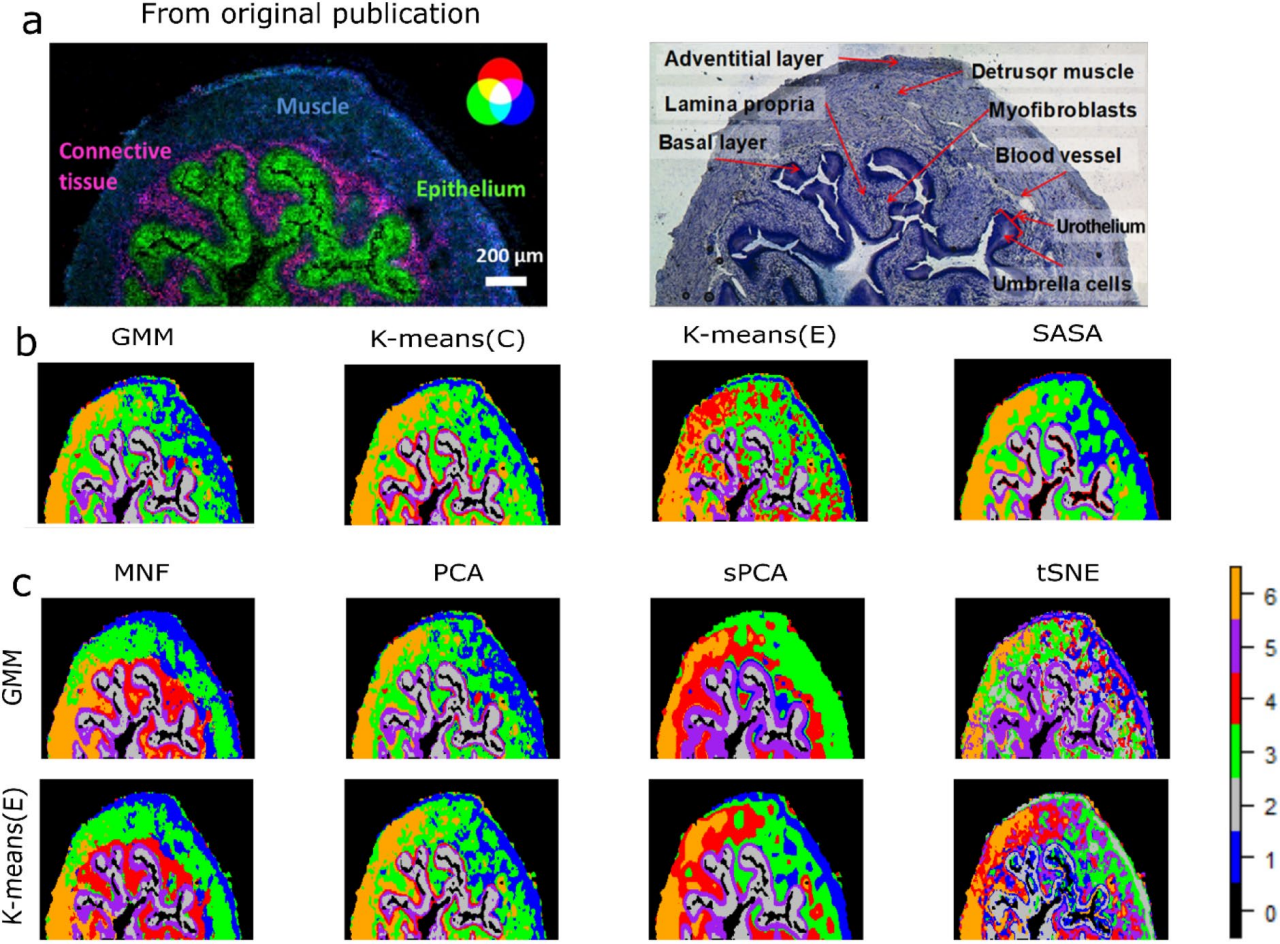
Clustering results from real MSI data. (**a**) A mouse urinary bladder image as published in the original publication. An overlay of three different molecular ions (blue: muscle tissue, green: urothelium, red: lamina propria) (left). An annotated optical image of a measured section stained with toluidine (right). (**b**) MSI data clustering using different clustering methods (second row). (**c**) MSI data clustering using GMM and Kmeans(E) cluster methods after reducing the original data dimension with different dimension reduction techniques.

Results from MSI data1: The real MSI data were clustered similarly to the synthetic data, i.e. the complete and reduced datasets. Both the Calinski-Harabasz index^59^ (CH) and the Davies-Bouldin index (DBI)^60^ internal cluster validity index suggested six/seven clusters for different combinations of clustering and dimension reduction methods. The six clusters were confirmed based on the observed anatomically different regions in the histology image (Fig. 6). All four clustering methods identified approximately similar types of clusters from the raw MSI data, except k-means(E) which further splits one particular cluster (4, (red). GMM and k-means(E) were applied to the reduced data obtained with the different dimension reduction methods. The clusters identified in the reduced dimensional space obtained by the MNF transform are smooth and continuous. The clustering methods give sub-optimal results after PCA since it misses certain important clusters such as cluster 4 which is observed in the MNF space (and corresponds to the lamina propria). The identified clusters in the sPCA space are approximately similar to the ones identified in the PCA space, but sPCA has over smoothened the data, resulting in an overlap of the clusters from different regions. The clusters identified in the tSNE space are over-segmented and heterogeneous.

The above-discussed results were obtained from MSI data binned with size 0.1 and preprocessed which results in data dimension 260 × 134 × 169. To see the impact of the increase in data dimension on clustering results, we analyzed similar MSI data in two different scenarios. In the first scenario, MSI data were binned with size 0.01 and preprocessed in a similar fashion which results in final data dimensions of 260 × 134 × 738. All clustering and dimension reduction methods are implemented on this newly processed MSI data. Overall, the clustering results and conclusions are similar (Supplementary Fig. S5) in low- and high-dimensional spaces except for the t-SNE. The clusters identified in the t-SNE space are better when we used low mass binning size or more molecular ions in our data. In the second scenario, MSI data were preprocessed using another peak-picking method called “simple” from the R-Cardinal package. Together with peak-picking, the remaining pre-processing steps (peak alignment, peak filter, mz alignment, mz bin, and filter) are performed using the Cardinal R package. The tolerance value for peak and mz alignment is set to 50 (in ppm). The total ion current (TIC) was performed to adjust the difference between spectra. A total of 9029 peaks were identified from the MSI data. The final data dimension is 260 × 134 × 9029. Unfortunately, all the results are not reproducible on this dataset. MNF transform failed on the MSI of very high dimensionality. On performing PCA and GMM clustering on a new dataset, the clustered image obtained is a slightly improved version of the image obtained with low-dimensional MSI data (Supplementary Fig. S8).

Results from MSI data2: The results are produced using the same set of clustering and dimension reduction techniques (Supplementary Fig. S7). The MSI data was clustered with five clusters (as proposed in the original article). In this case, we do not have any stained image to compare the identified clusters, but overall clustering results and conclusions are similar as derived from previous MSI data.

### Impact of number of dimensions on clustering results

The clustering analysis was performed on a reduced number of dimensions derived from different unsupervised methods. In our research, all the simulations were performed with the fixed input parameter value (Table 1). The reduced data obtained from PCA and sPCA have a dimension of size four, as in the majority of scenarios, this number of components explains more than 80% of the total variance. This is also confirmed by a method developed to select the number of components from PCA^61^. In the case of the MNF transform six components were selected following the exact approach used in the original publication^54^. However, in a small simulation study (results are not shown here), we noticed similar results can be achieved using four components from the MNF transform. The two t-SNE dimensions were used for cluster analysis. This was a choice made since there is no intuitive way to select t-SNE dimensions.

The poor clustering results were observed with t-SNE, both for synthetic and real MSI data. To cross-validate the impact of t-SNE dimensions and perplexity parameter value on clustering, in real MSI data we repeat the analysis where GMM clustering was performed on t-SNE space consisting of a different number of dimensions and perplexity parameter value (Supplementary Fig. 6). The results showed clustering on t-SNE two-dimension space with perplexity equal to 5, and 50 give similar results as clustering on MNF space. And, if we consider t-SNE three-dimensional space, similar results were obtained with perplexity parameters 10 and 30. In t-SNE three-dimensional space, the over-segmented clustered image is obtained when perplexity parameter is equal to 5 and the over-smooth image when the perplexity parameter is equal to 10. The results from t-SNE are quite sensitive to input parameters used and how one can select the right parameter for cluster analysis required further analysis.

## Discussion

Very often a clustering method works well on some datasets but may perform poorly in other datasets, owing to different data structures and characteristics. In this paper, the performance of four clustering methods was investigated on simulated and real MSI data. To our knowledge, this is the first time, the evaluation of clustering methods was performed by simulated multivariate spatially autocorrelated data. Our simulation results on synthetic data show that each clustering method has its limitations and with an increase in data complexity, the performance of clustering methods gradually decreases.

The real MSI data have multiple spatial structures and variables with different distributions and intensities (Fig. 3). Therefore, our simulation results from spatial data type three, mixture distribution, and VarMod_10 must be most representative of real MSI data. However, all simulations shown are equally important to better understand the performance of these methods on a variety of datasets. For example, to understand how easily these methods can identify the clusters with different spatial structures irrespective of the difference in cluster means, we start with a scenario where all variables have zero mean and gradually increase the mean of variables’ intensity per cluster. This simulation was designed considering MSI data from low- and high-resolution instruments or sometimes due to the ion suppression effect ions from certain tissue have relatively low intensity. Our simulation results are shown for complex spatial data, clustering methods GMM and k-means (C) give good performance if at least 10 variables in the data are modified. In addition, we also tested the performance of clustering methods in the presence of noisy/non-spatial variables. Here, we tried to mimic the matrix peaks in MSI data which does not follow any spatial structure. In our study, we did not any difference in the results in the presence of those variables. However, the simulation was performed only with 5 noisy variables, probably with more noisy variables in the data clustering results get affected. All simulations were repeated with synthetic data with two and four clusters, to understand the impact of cluster size on clustering results. And we found our clustering results were quite consistent on both types of synthetic data.

It is not uncommon to perform clustering of MSI data after preprocessing the data using dimension reduction or transformation techniques^16,18,54^. The dimension reduction or transformation techniques represent the data in a few dimensions space which makes cluster identification easy. However, this also makes clustering results dependent on the type of dimension reduction technique and the number of final dimensions used in cluster analysis. In our paper, the performance of four different dimension reduction techniques in combination with clustering methods was tested. The t-SNE is one most frequently used data transformation techniques with MSI data. In our simulation study, poor results were obtained with t-SNE. There are certain limitations or disadvantages regarding t-SNE which make it less feasible for cluster analysis. The results obtained from t-SNE are highly dependent on random data generation points and the right perplexity parameter (also shown with real MSI data Supplementary Fig. S6). And there is no easy way to find the right perplexity parameter. Another main challenge with the t-SNE is computational complexity. In our paper, we used the R package Rtsne for the implementation of the t-SNE method. The following R-package uses reduced PCA dimensions as an input to make the process computationally faster and returns the maximum t-SNE three dimensions. The analysis using Rtnse makes our simulation study slightly sub-optimal as we can not use more than three t-SNE dimensions. However, the original t-SNE algorithm produced results with data of the size used in this paper in many hours which makes the simulation study even harder.

Apart from the type of dimension reduction technique, the clustering results are also found to be sensitive to the dimensions of the MSI dataset. The clustering results from PCA and GMM on high-dimension MSI are close to the clustering results from MNF and GMM. Overall, the best clustering results were obtained after preprocessing the data with MNF transform before clustering analysis. However, the results apply to the MSI data of restricted dimensions. The MNF transform cannot be performed with data of very high dimensions. In the original article, the author used the signal to noise ratios to limit the number of dimensions for a reasonable covariance matrix calculation.

## Conclusion

In this study, we had shown the limitations and strengths of different unsupervised methods for the extraction of spatially relevant patterns from MSI data. The simulation results from synthetic data have shown that the performance of clustering methods declined with an increase in complexity in the spectral and spatial domain. The dimension reduction techniques help clustering methods to identify relevant clusters. The clustering methods GMM and k-means(E) give high adjusted rand index values with reduced data obtained after MNF transformation. However, the results shown in this paper are only applicable to the preprocessed MSI data with a restricted number of dimensions.

## Supplementary Information

Supplementary Information.

## Data Availability

The MSI datasets used in this study are publicly available from PRIDE repository (PXD001283) and GigaScience Repository (see above “Material and Methods” section).
