# Supplementary material for: Evaluation and comparison of unsupervised methods for the extraction of spatial patterns from mass spectrometry imaging data (MSI)
Source: Sci Rep. 2022 Sep 20;12:15687. doi: 10.1038/s41598-022-19365-4 (PMC9489880; doi:10.1038/s41598-022-19365-4)
Supplement: Supplementary file 1 — Supplementary Information. [file 41598_2022_19365_MOESM1_ESM.docx]

**Supplementary Material :**

**Evaluation and comparison of unsupervised methods for the extraction of spatial patterns from mass spectrometry imaging data (MSI)**

Mridula Prasad^1,2^, Geert Postma^1*^, Pietro Franceschi^2^, Lutgarde M.C. Buydens^1^, Jeroen Jansen^1^

^1^IMM/ Analytical Chemistry, Radboud University, Heyendaalseweg, 6525 AJ Nijmegen, Netherlands.

^2^Unit of Computational Biology, Research and Innovation Center, Fondazione Edmund Mach, 38010, San Michele all’ Adige, Italy.

Corresponding Author

* E-mail: [chemometrics@science.ru.nl](mailto:chemometrics@science.ru.nl)

Supplementary Text S1: Brief description of unsupervised methods tested in our study.

**Clustering methods**

1. K-means with Euclidean distance (k-means (E)): the clustering method assigns each pixel to a predefined number of classes using the Euclidean distance as a dissimilarity measure. The higher the distance implies low the similarity.
2. K-means with correlation distance (k-means(C)): the clustering method relies on correlation distance instead of Euclidean distance to assign the pixels to the class with the highest similarity. The correlation distance between two points is one minus the Pearson correlation between two points (treated as vectors).
3. Gaussian mixture modeling (GMM): GMM clustering method assumes the observed data generated from a mixture of different probability distributions which can be identified as individual clusters. The cluster membership of an observation can be derived from the model parameters (mean and covariance matrix). This approach is more flexible than the standard heuristic methods as it allows clusters of different volumes and shapes and can account for within-cluster correlations between the variables. However, GMM on high-dimension data such as MSI could fail to cluster the data accurately due to many free parameters in the covariance matrices. Therefore, GMM is performed on reduced data which is derived using principal component analysis (PCA). The dimension of reduced data obtained from PCA is equal to the number of principal components explaining at least 80% of the total variance.
4. Spatially aware structurally adapted (SASA) k-means clustering: is a spatial adapted version of the k-means (E) where the dissimilarity matrix is modified to take into account spatial relationships between pixels.

**Dimension reduction techniques**

1. Principal component analysis (PCA): PCA works by projecting the data onto orthogonal bases that explain the maximum amount of variance in the data. The first base points in the direction of maximal variance in the data. The next, orthogonal base point in the direction of remaining maximal variance and so on.

2. Spatial principal component analysis (sPCA): sPCA is the modified version of the PCA that takes into account spatial autocorrelation. sPCA does the matrix factorization like traditional PCA, where the variance-covariance matrix is derived from the original data matrix and its spatially weighted version. For spatial PCA, a spatial weight matrix is required to show the association between observations. A spatial weight matrix is a two-dimension matrix, where the observations are connected and receive one value else zero. For sPCA, the spatial weight matrix was created using spdep R package where the range of spatial autocorrelation was set to ten pixels.

3. Minimum noise fraction (MNF) transform: MNF transform is similar to the PCA which maximizes the signal-to-noise ratio. The signal covariance matrix is the covariance matrix from the complete dataset. And, for the noise covariance matrix, first noise data are estimated by fitting a linear or any other type of function in a moving window passing through each observation/pixel in the data. The MNF method orders the components in terms of image quality. Therefore, by utilizing the last few components, a new dataset with all cleaned images can be achieved.

4. t-Distributed Stochastic Neighbor Embedding (t-SNE): t-SNE is a non-linear dimension reduction method that tries to preserve the local and global structure in the lower two-dimensional space. t-SNE is implemented with Barnes-Hut approximation after initial dimension reduction using PCA.





Supplementary Figure1: **Schematic representation of synthetic data generation steps.** a) Two types of correlation matrices are used to simulate multivariate spatially autocorrelated data. The metabolic correlation matrix is used to add the correlation between variables and the spatial covariance matrix, for adding spatial autocorrelation between the nearby observations. b) A method for creating a spatial covariance matrix. First, a separate cluster-specific spatial covariance matrix was created with the help of the distance matrix and spherical covariance function. Later, all individual covariance matrices were combined into a single final spatial covariance matrix.

**Spatial data 1**

Non-spatial a b Spatial a b

**
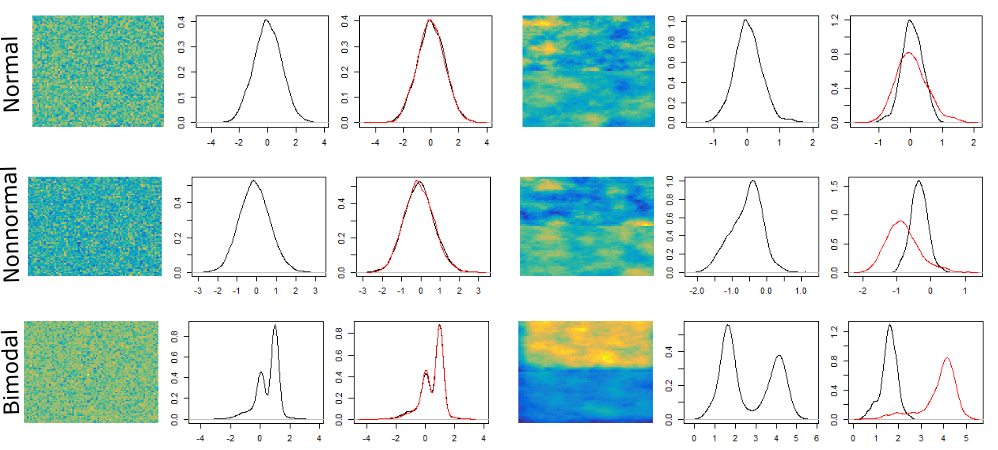
**

**Spatial data 2**

Non-spatial a b Spatial a b
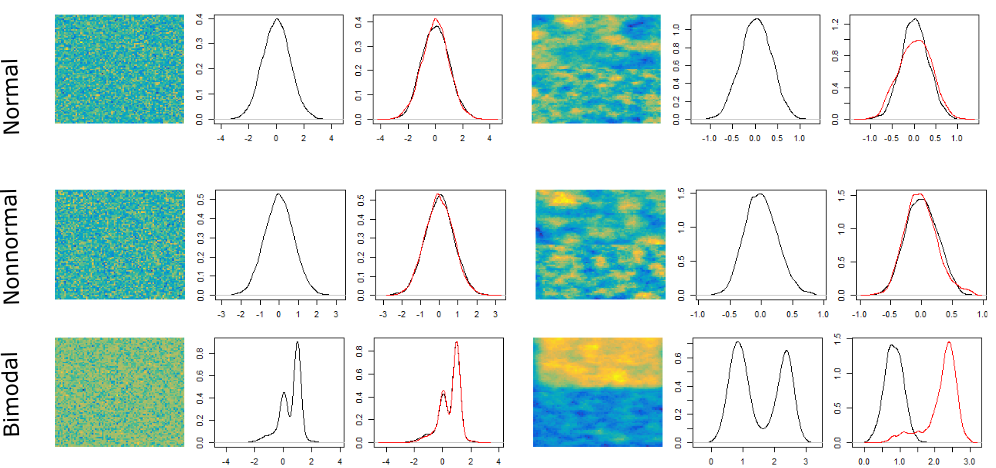


**Spatial data 3**

Non-spatial a b Spatial a b


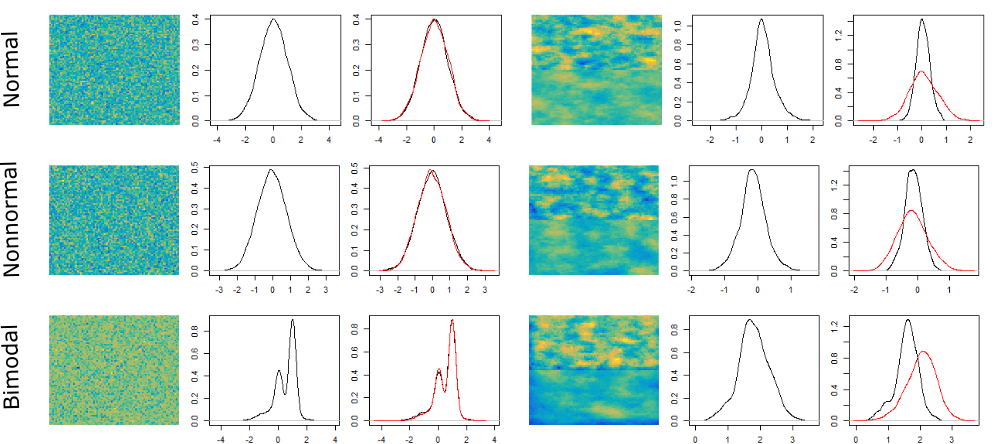


Supplementary Figure S2: **The density plots of a single example variable from non-spatial (left) and spatial (right) data (type =1,2,3).** The density plot is created first by selecting the observations from the complete image (a) and then from individual clusters (b).

Supplementary Table S1: Spatial parameters used to simulate synthetic data for four cluster problem

| Spatial data | Cluster1 |  | Cluster2 |  | Cluster3 |  | Cluster4 |  |
| --- | --- | --- | --- | --- | --- | --- | --- | --- |
| Parameters | Range1 | Sill1 | Range2 | Sill2 | Range3 | Sill3 | Range4 | Sill4 |
| 1 | 20 | 0.1 | 20 | 0.2 | 20 | 0.3 | 20 | 0.4 |
| 2 | 10 | 0.1 | 20 | 0.1 | 15 | 0.1 | 25 | 0.1 |
| 3 | 10 | 0.1 | 20 | 0.2 | 15 | 0.3 | 25 | 0.4 |


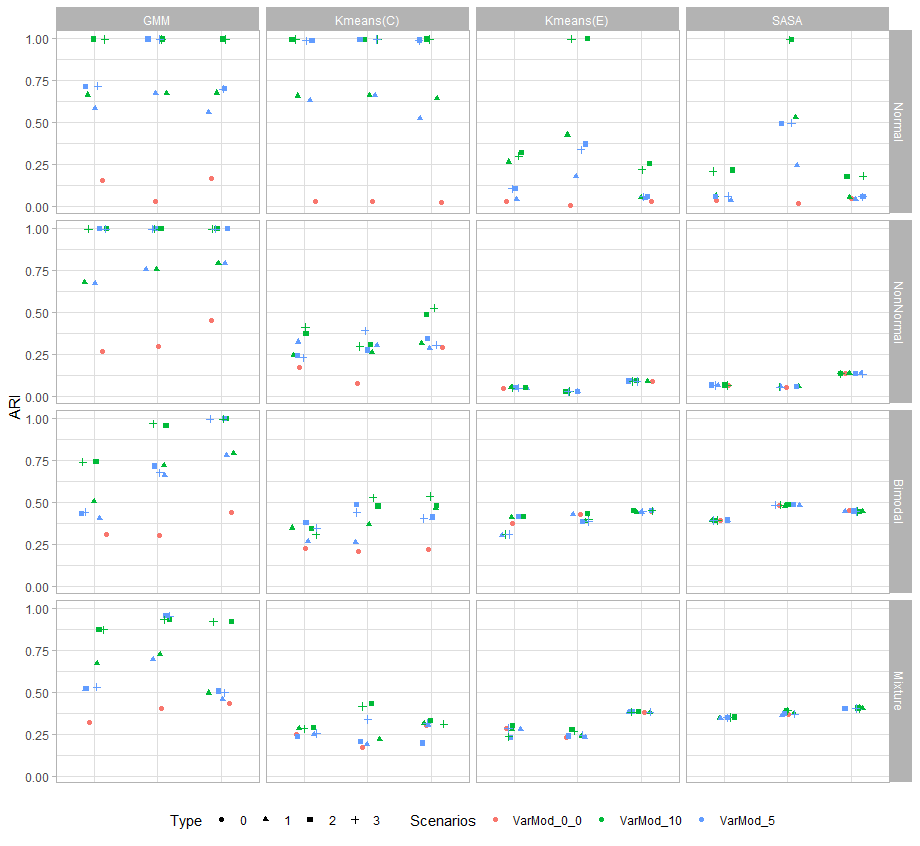


Supplementary Figure S3: **A plot of simulation results for the flour clusters problem**. All four different clustering methods (GMM, K-means(C), K-means(E), and SASA) are tested on synthetic data generated by varying statistical and spatial parameters. The performance of clustering methods is monitored based on their adjusted rand index (ARI) value. In the above figure, the ‘Scenarios’ represent the number of variables with updated mean values in synthetic data, such as VarMod_0: all variables in synthetic data with zero mean; VarMod_10: 10 variables in synthetic data with mean value 0.5; VarMod_5: 5 variables in synthetic data with mean value 0.5. And the ‘Type’ represents the nested conditions with scenarios (0: all variables with mean 0; 1: variables with updated means in single cluster (VarMod_0_5, VarMod_0_10); 2: variables with updated means in both clusters (VarMod_5_5, VarMod_10_10); 3: simulation with added noise (VarMod_5_5_addnoise, VarMod_10_10_addnoise). The statistical and spatial parameters of spatial Data 1, 2, and 3 are mentioned in Supplementary Table S1.


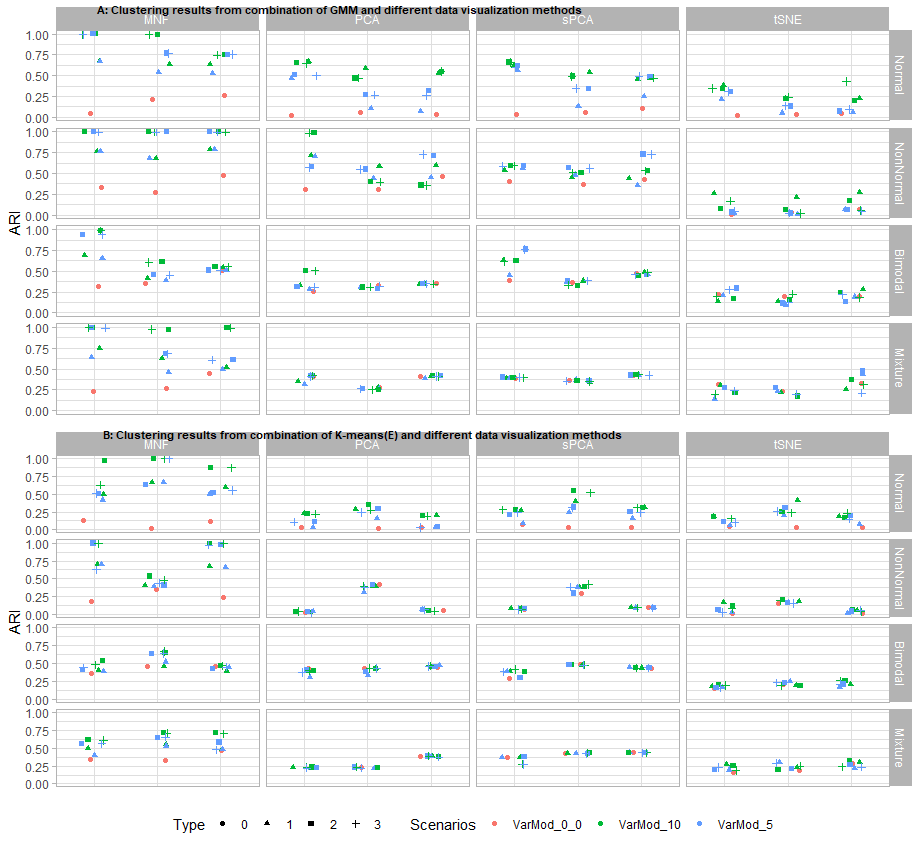


Supplementary Figure S4: **A plot of simulation results from a combination of dimension reduction techniques and clustering methods.** Four different dimension reduction techniques (MNF, PCA, sPCA, and t-SNE) were implemented on synthetic data before cluster analysis. Cluster analysis was performed with a) GMM and, b) Kmeans(E) methods. The performance of clustering methods is monitored based on their adjusted rand index (ARI) value. In the above figure, the ‘Scenarios’ represent the number of variables with updated mean values in synthetic data, such as VarMod_0: all variables in synthetic data with zero mean; VarMod_10: 10 variables in synthetic data with mean value 0.5; VarMod_5: 5 variables in synthetic data with mean value 0.5. And the ‘Type’ represents the nested conditions with scenarios (0: all variables with mean 0; 1: variables with updated means in single cluster (VarMod_0_5, VarMod_0_10); 2: variables with updated means in both clusters (VarMod_5_5, VarMod_10_10); 3: simulation with added noise (VarMod_5_5_addnoise, VarMod_10_10_addnoise). The details about the statistical and spatial parameters modified are given in Table 1 and Supplementary Table S1.


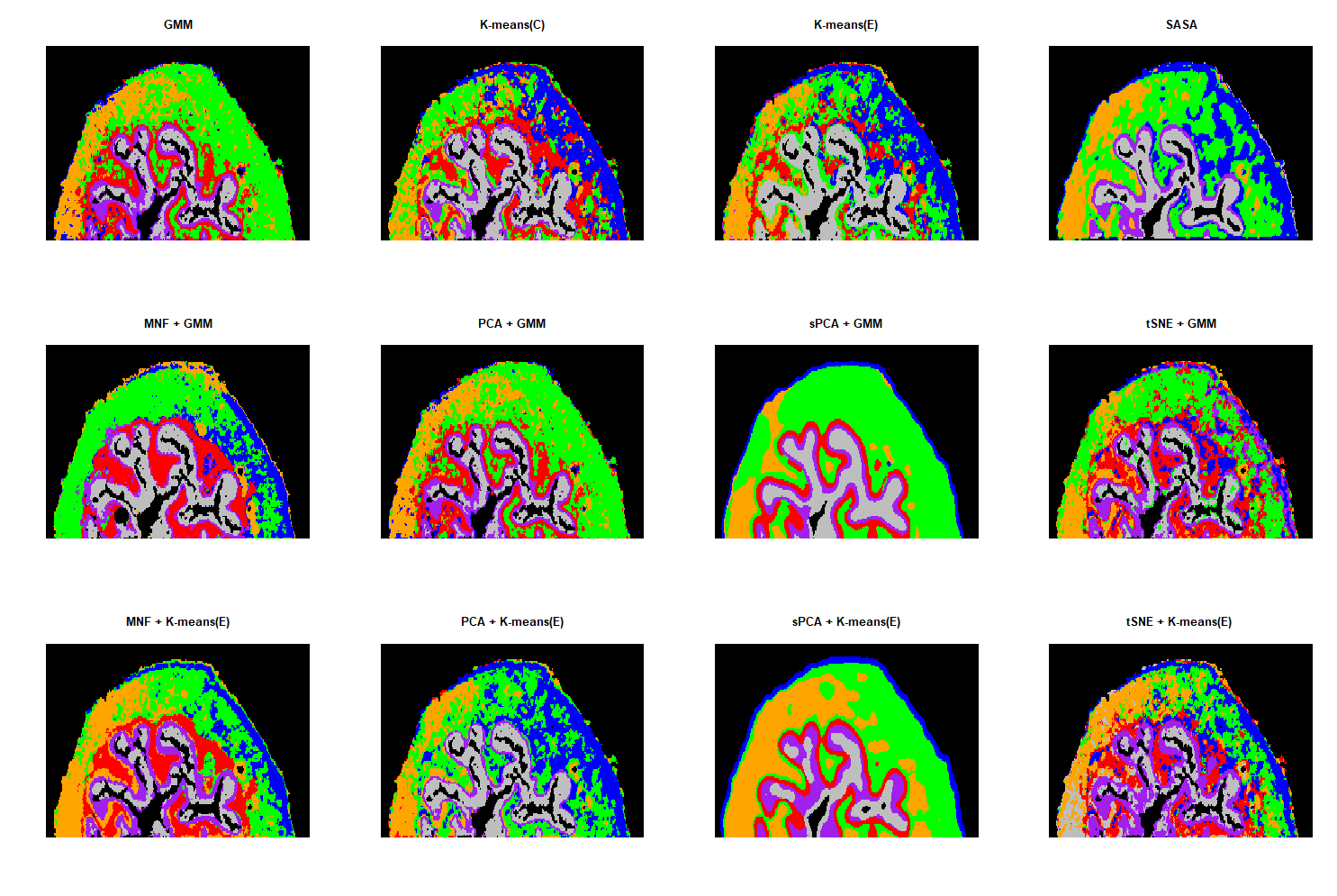


Supplementary Figure S5: **Clustering results from real MSI data (mass bin size = 0.01Da)**


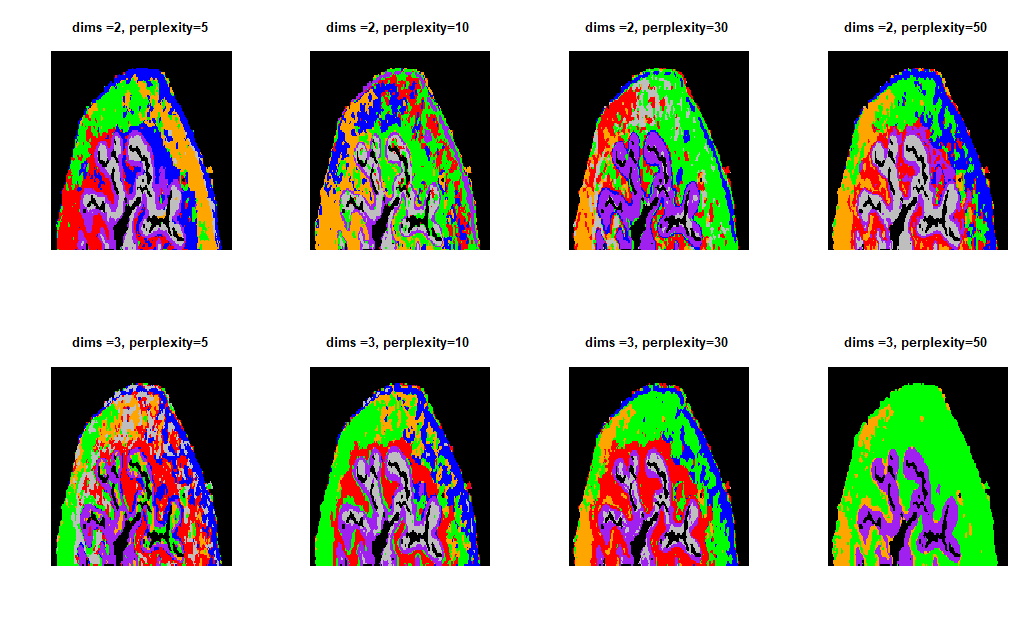


**Supplementary Figure S6: Clustering results from real MSI data with different dimensions of t-SNE together with different input parameter (perplexity) values in t-SNE.** The GMM clustering method is used after applying the t-SNE technique with different input parameters on the MSI data.


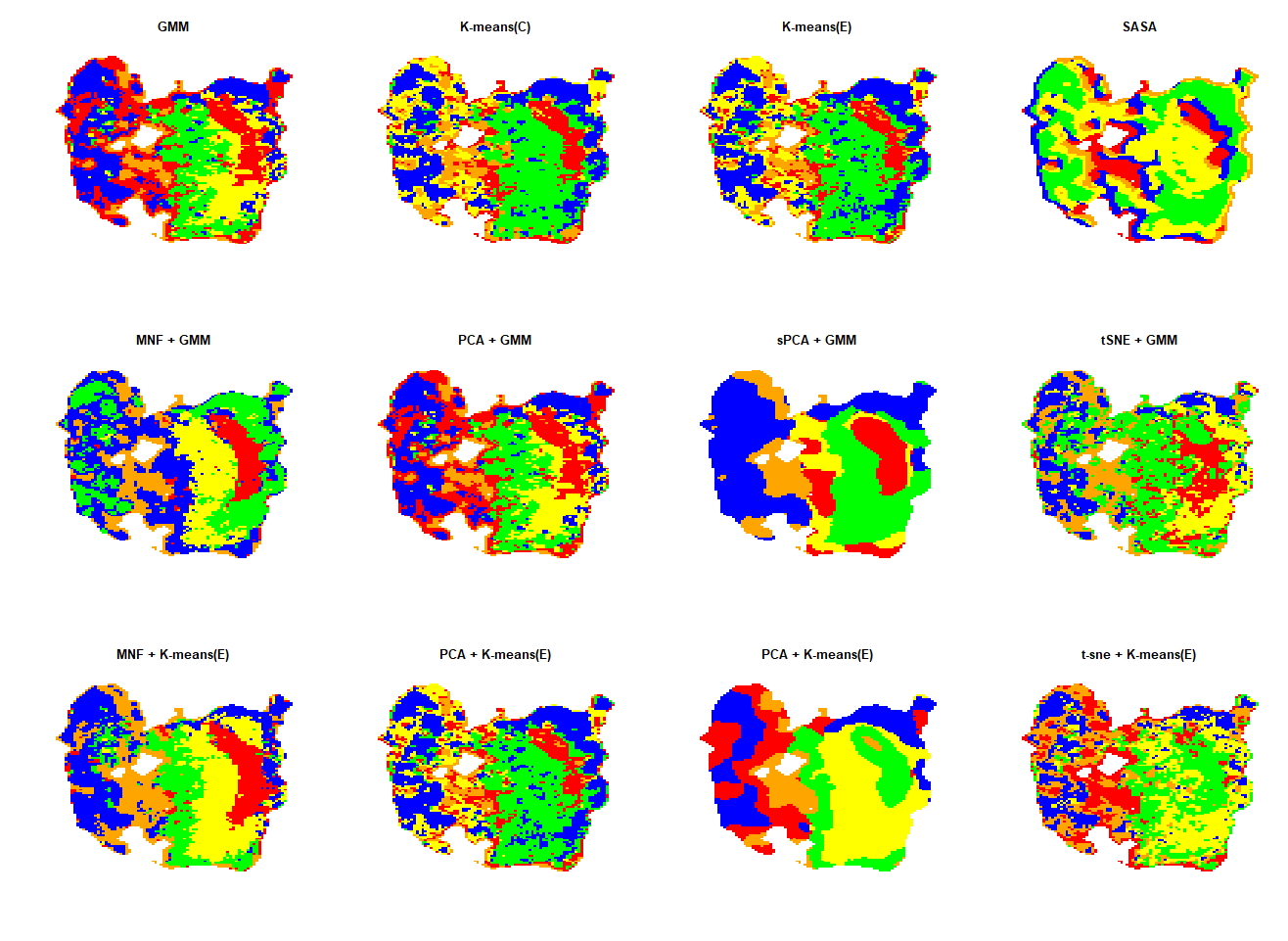


**Supplementary Figure S7: Clustering results from MALDI-TOF tumor MSI data.**


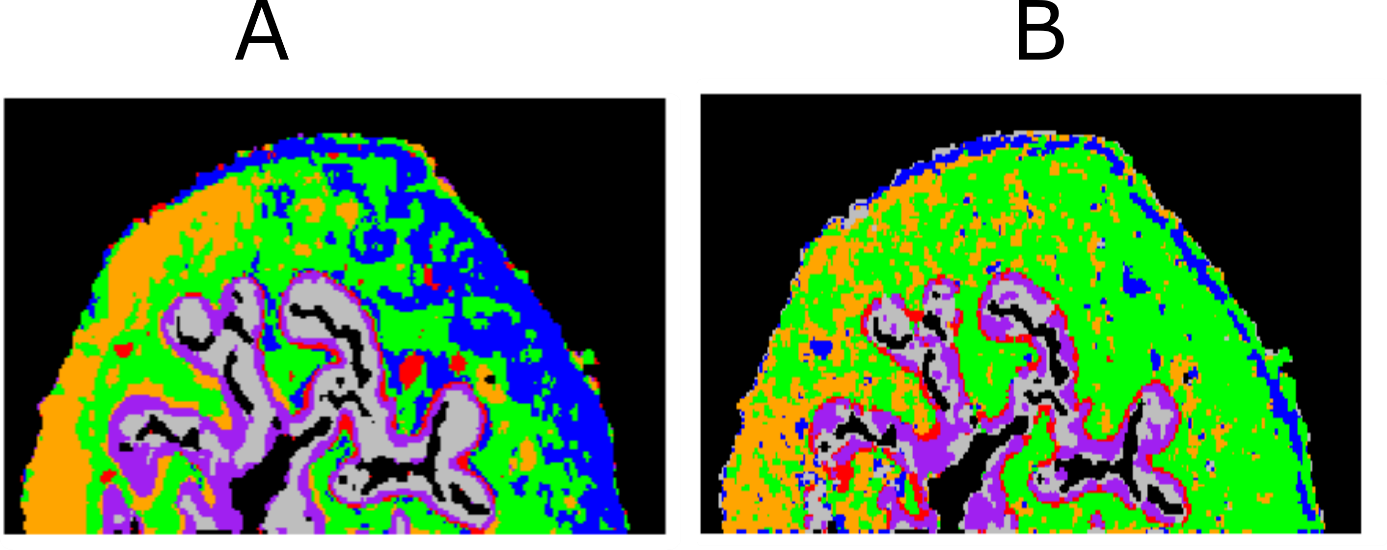


**Supplementary Figure S8**: **Clustering of MSI data with GMM clustering method on the four PCA dimensions.** The clustered image obtained from the A: initial preprocessed data ( total peaks = 169) B: after peak picking with the ‘simple’ method (total peaks = 9029).

### R-code for generation of two clusters spatially autocorrelated data and evaluation of clustering methods

suppressMessages(library(SimMultiCorrData))

suppressMessages(library(MCMCglmm))

suppressMessages(library(mzImage))

## medianFilterR: Function to perform median filtering on 2-D image

medianFilterR <- function(sampleMat)

{

B = matrix(0, nrow= dim(sampleMat)[1],ncol=dim(sampleMat)[2])

modifyA = matrix(0,nrow=(dim(sampleMat)[1]+2),ncol=(dim(sampleMat)[2]+2))

for(x in 1:dim(sampleMat)[1])

{

for(y in 1:dim(sampleMat)[2])

{

modifyA[x+1,y+1] = sampleMat[x,y] } }

for(i in 1:(dim(modifyA)[1]-2))

{

for(j in 1:(dim(modifyA)[2]-2))

{

window = rep(0,9)

inc = 1

for(x in 1:3)

{

for(y in 1:3)

{

window[inc] = modifyA[i+x-1,j+y-1]

inc = inc +1

} }

med = sort(window)

B[i,j] = med[5]

} }

return(B)

}

## simcor : Function to add desired correlation between variables

# this function simulates a block correlation matrix

# The size and base correlation for each block is user specified

# There is an additional delta parameter for the off diagonal correlations

# k is the number of groups

# size is a vector of length k specifying the size of each group

# rho is a vector of length k specifying base correlation values

# epsilon <- 0.99 - max(rho)

# eidim is the space from which the noise is generated, the smaller the more noise

# delta is the correlation of the off diagonal blocks

simcor = function (k = 6, size = c(10, 5, 8, 2, 15, 50), rho = c(0.7, 0.7, 0.5, 0.9, 0.85, 0.4), delta = 0.39, epsilon = 0.99 - max(rho), eidim = 2)

{

ndim <- sum(size)

bigcor <- matrix(rep(delta, ndim * ndim), ncol = ndim)

for (i in 1:k) {

cor <- matrix(rep(rho[i], size[i] * size[i]), ncol = size[i])

if (i == 1) {bigcor[1:size[1], 1:size[1]] <- cor}

if (i != 1) {bigcor[(sum(size[1:(i - 1)]) + 1):sum(size[1:i]),

(sum(size[1:(i - 1)]) + 1):sum(size[1:i])] <- cor}

}

diag(bigcor) <- 1 - epsilon

eivect <- c()

for (i in 1:ndim) {

ei <- runif(eidim, -1, 1)

eivect <- cbind(eivect, sqrt(epsilon) * ei/sqrt(sum(ei^2)))

}

bigE <- t(eivect) %*% eivect

cor.nz <- bigcor + bigE

cor.nz

}

## Covmatrix : Function to create a spatially autocorrelated covaraince matrix

## Calculate distance matrix with range parameter

distmatrix <- function(dat = as.matrix(dat),rng1=5)

{

n=dim(dat)[1]

dist.mat1 = matrix(0, nrow = dim(dat)[1], ncol = dim(dat)[1])

for(i in 1:n){

for(j in 1:n){

dist.mat1[i,j] = sqrt(((dat[i,1] - dat[j,1])^2) + ((dat[i,2] - dat[j,2])^2))

if(dist.mat1[i,j] > rng1) {dist.mat1[i,j] = rng1}

dist.mat1[j,i] = dist.mat1[i,j]

}

}

return(dist.mat1)

}

#Calculating the cubed distances used in the spherical covariance function

CubeDistance <- function(dist.mat1 = dist.mat1)

{

n = dim(dist.mat1)[1]

dist.mat1.3 = matrix(0, nrow = dim(dist.mat1)[1], ncol = dim(dist.mat1)[1])

for(i in 1:n){

for(j in 1:n){

dist.mat1.3[i,j] = dist.mat1[i,j]^3

dist.mat1.3[j,i] = dist.mat1.3[i,j]

} }

return(dist.mat1.3)

}

#Calculating the variance-covariance matrix

Covmatrix <- function(dat = as.matrix(dat),rng1=5,sill1=1)

{

n=dim(dat)[1]

dist.mat1 = distmatrix(dat,rng1)

dist.mat1.3 = CubeDistance(dist.mat1)

c1 = -1.50*(sill1/rng1)*dist.mat1

c2 = 0.50*(sill1/rng1^3)*dist.mat1.3

c3 = matrix(sill1,n,n)

c4 = c1 + c2 + c3

L1 = chol(c4)

return(c4)

}

# SimulateDensityData : Simulate data from normal (Type ='N'), bimodal distribution (Type ='M'), #non-normal data (Type ='NN'), or combination of all (Type = 'A')

MixtureDensityData <- function(N =6400)

{

U =runif(N);rand.samples = rep(NA,N)

for(i in 1:N){

if(U[i]<.3){

rand.samples[i] = rnorm(1,0,1)

}else if(U[i]<.8){

rand.samples[i] = rnorm(1,1,0.2)

}else{

rand.samples[i] = rnorm(1,0.05,0.2)

} }

return(rand.samples)

}

### Acceptable kurtosis and skewness value for non-normal distribution according to Fleishman algorithm

x <- seq(0,2.4,0.2); y <- seq(-2,10,0.5)

sk <- expand.grid(skewness =x, kurtosis = y)

skew = sk[,1]; kurt = sk[,2];

idx = which(kurt > (-1.2264489 + 1.6410373* skew^2));

sk = sk[idx, ];

SimulateDensityData <- function(Type="N",sk)

{

if(Type == 'N'){

sData = matrix(rtnorm(variables*Pixels,0,1,-1,1), nrow=100, ncol=6400) ;

sData = t(sData)

}else if(Type=='M'){

FinalData <- MixtureDensityData(N=6400)

}else if(Type =='NN')

{

NonNormalData <- matrix(0,nrow=6400,ncol=100)

for(i in 1:100)

{

id1 <- sample(seq(1,214,1),1);

c<-find_constants("Fleishman", sk[id1,1], sk[id1,2])

N <- nonnormvar1("Fleishman", c$constants[[1]], c$constants[[2]], c$constants[[3]], c$constants[[4]],n=6400 )

NonNormalData[,i] <- N$continuous_variable$V1

}

FinalData <- NonNormalData

} else if(Type == 'A')

{

AllCombineData <- matrix(0,nrow=6400,ncol=100)

id <- sample(1:100)

tmpData <- matrix(rtnorm(60*6400,0,1,-1,1), nrow=60, ncol=6400)

tmpData <- t(tmpData)

AllCombineData[,id[1:60]] <- tmpData

for(i in 61:80) AllCombineData[,id[i]] <- MixtureDensityData(N=6400)

for(i in 81:100)

{

id1 <- sample(seq(1,214,1),1);

c<-find_constants("Fleishman", sk[id1,1], sk[id1,2])

N <- nonnormvar1("Fleishman", c$constants[[1]], c$constants[[2]], c$constants[[3]], c$constants[[4]],n=6400 )

AllCombineData[,id[i]] <- N$continuous_variable$V1

}

FinalData <- AllCombineData

}

return(FinalData)

}

**## Evaluation of clustering methods on spatial autocorrelated data with two clusters**

#### Create enviornment for Parallel Computing

clusterExport(cl, c("ClusterAnalysis","RunClusteringMethods","Covmatrix",'simul','distmatrix','CubeDistance','rnorm2','D',"medianFilterR","%!in%","imgvalue","variables",

"simcor",'simul','SupervisedClustering',"MixtureDensityData","sk"), envir=environment())

init = clusterEvalQ(cl, { library(amap);library(ade4);library(MCMCglmm);library(fpc);library(viridis);library(clValid);library(clusterSim);library(fields);library(Cardinal);library(mclust); library(tsne);library(cluster);library(reshape2);

library(ggplot2);library(gridExtra); library(SimMultiCorrData);NULL })

no_cores <- detectCores() - 1

cl <- makeCluster(no_cores)

### DEFAULT PARAMETERS

simul = 100; RandFinal = list(); WssFinal= list()

variables = 100; Pixels=6400;metCor=0.5;rho =0.5;

imgvalue = c(rep(1,3200),rep(2,3200))

id = which(imgvalue %in% 1);id1 = which(imgvalue %in% 2);countf=1

SpatialPars = matrix(c(20,20,0.1,0.3,

10,20,.1,0.1,

20,10,0.1,0.3),nrow = 3,byrow = T)

### Add spatial anisotropy: shift original spatial co-ordinates by angle of 45

xycoord <- expand.grid(1:80,1:80)

xycoord = as.matrix(xycoord)

psiA = 45

rm <- matrix(c(cos(psiA), -sin(psiA), sin(psiA), cos(psiA)), ncol = 2)

tm <- diag(c(1, 1/2))

coords.mod <- xycoord %*% solve(rm %*% tm)

xycoord = coords.mod

dat = xycoord[id,];dat1 = xycoord[id1,]

### REQUIRED FUNCTIONS

'%!in%' <- function(x,y)!('%in%'(x,y))

## Simulations with different clustering methods

SupervisedClustering <- function(sData)

{

coord1 = expand.grid(x=1:80,y=1:80);

RandMatrix = matrix(0,nrow=1,ncol=5)

Sampledata = seq(1,dim(sData)[1],5)

print("----------------------------------------------------Data clustering in process----------------------------------------------------------------------")

#1 Clustering method : Kmeans with Euclidean distance

cluster1 = kmeans(scale(sData,scale=FALSE),2);Kmean_eud= cluster1$cluster; RandMatrix[1,1] = adjustedRandIndex(Kmean_eud,imgvalue);

#2 Clustering method : Kmeans with Correlation distance

cluster2 <- Kmeans(scale(sData,scale=FALSE), 2,method="correlation",iter.max = 150);Kmean_corr = cluster2$cluster;dim(Kmean_corr) = c(80,80);Kmean_corr = medianFilterR(Kmean_corr);Kmean_corr[Kmean_corr==0]=1;

RandMatrix[1,2] = adjustedRandIndex(as.vector(Kmean_corr),imgvalue);

#3 Clustering method : GMM on PCA space

pca <- prcomp(sData,center = TRUE);cluster3 = Mclust(pca$x[,1:5],2,iter.max=150,initialization = list(subset=Sampledata));clut_gmmp = cluster3$classification;dim(clut_gmmp) = c(80,80);clut_gmmp = medianFilterR(clut_gmmp);

clut_gmmp[clut_gmmp ==0]=1;RandMatrix[1,3] = adjustedRandIndex(as.vector(clut_gmmp),imgvalue);

#4 Clustering method: spatial kmeans Theodore version

sset <- SImageSet(data=t(scale(sData,scale=FALSE)), coord=coord1);

cluster4 <- spatialKMeans(sset, r=c(3), k=2);skm_img = as.numeric(cluster4$cluster[[1]]);RandMatrix[1,4] = adjustedRandIndex(skm_img,imgvalue);

#5 Clustering method : spatial k-means sasa

cluster5 <- SImageSet(data=t(scale(sData,scale=FALSE)), coord=coord1);skm <- spatialShrunkenCentroids(sset, r=c(3), k=2,s=2);skm_img1 = as.numeric(skm$classes[[1]]);RandMatrix[1,5] = adjustedRandIndex(skm_img1,imgvalue);

return(RandMatrix)

}

## Perform clustering on synthetic data

RunClusteringMethods <- function(D,variables=100,Pixels=6400)

{

sData <- SimulateDensityData(Type="A",sk)

CorMat = simcor(4,size = c(20,50,20,10),rho =c(0.7,0.5,0.6,0.4),eidim = 100)

L = chol(CorMat) ; r = sData %*% L

correlatedvariable <- function(x,D)

{x = t(x) %*% D ;x =as.vector(t(x))}

CorrelatedVariables <- matrix(apply(r,2,function(x)correlatedvariable(x,D)),nrow = Pixels,ncol=variables)

sData = CorrelatedVariables

sData1 = apply(sData,2,function(x)x+rnorm2(6400,sample(seq(0.1,0.2,0.01),1),0.1))

sData1 <- sData1 + abs(min(sData1))

sData <- sData1;

id_noise <- sample(c(1:100),10);

id_remain <- setdiff(c(1:100),sample(c(1:100),10)); id_remain <- sample(id_remain)

CorrelatedVariables <- sData

RandMatrix = matrix(0,nrow=7,ncol=5);

# 1 with original data

RandMatrix[1,] = SupervisedClustering(sData) ;

# 2 increase mean for 5 variables in first cluster

id1 = id_remain[1:5]

sData[1:3200,id1] = sData[1:3200,id1]+rep(rnorm2(3200,0.5,0.1),length(id1));

RandMatrix[2,] = SupervisedClustering(sData) ;

# 3 increase mean with 1 for 5 variables in another cluster

id2 = id_remain[5:10]

sData[3201:6400,id2] = sData[3201:6400,id2]+rep(rnorm2(3200,0.5,0.1),length(id2));

RandMatrix[3,] = SupervisedClustering(sData) ;

# 4 add noisy variables

sData = cbind(sData, matrix(rnorm2(3200,0.5,0.1),nrow = Pixels,ncol=5));

RandMatrix[4,] = SupervisedClustering(sData) ;

# 5 increase mean with 1 for 10 variables in first clusters

sData = CorrelatedVariables

id1 = id_remain[1:10]

sData[1:3200,id1] = sData[1:3200,id1]+rep(rnorm2(3200,0.5,0.1),length(id1));

RandMatrix[5,] = SupervisedClustering(sData) ;

# 6 increase mean with 1 for 10 variables in second cluster

id2 = id_remain[11:20]

sData[3201:6400,id2] = sData[3201:6400,id2]+rep(rnorm2(3200,0.5,0.1),length(id2));

RandMatrix[6,] = SupervisedClustering(sData) ;

# 7 add noisy variables

sData = cbind(sData, matrix(rnorm2(3200,0.5,0.1),nrow = Pixels,ncol=5));

RandMatrix[7,] = SupervisedClustering(sData) ;

output = list(RandMatrix)

return(output)

}

## Function to generate spatial covariance matrix and call other clustering functions

ClusterAnalysis <- function(x,simul=1){

gc(reset=TRUE)

print(paste("r1",x[1],"r2",x[2],"s1",x[3],"s2",x[4],sep="="))

sig1 = Covmatrix(dat,x[1],x[3]);sig2 = Covmatrix(dat1,x[2],x[4]); L1 = chol(sig1);L2 = chol(sig2)

sig12 = rho*t(L1)%*%L2 ;sig21 = rho*t(L2)%*%L1

sig.row1 = cbind(sig1,sig12) ;sig.row2 = cbind(sig21,sig2) ;sig = rbind(sig.row1,sig.row2)

D = chol(sig)

ClusterResults <- parallel::parSapply(cl,1:simul, function(i)RunClusteringMethods(D))

RandMatrix <- do.call(rbind,ClusterResults)

output = list(RandMatrix)

save(RandMatrix,file=paste(paste("MixtureDensityData","r1",x[1],"r2",x[2],"s1",x[3],"s2",x[4],sep="_"),".RData",sep=""))

return(output)

}
